# Characterization of Dielectric Nanocomposites with Electrostatic Force Microscopy

**DOI:** 10.1155/2017/4198519

**Published:** 2017-09-25

**Authors:** D. El Khoury, V. Fedorenko, J. Castellon, M. Bechelany, J.-C. Laurentie, S. Balme, M. Fréchette, M. Ramonda, R. Arinero

**Affiliations:** ^1^Institut d'Electronique et des Systèmes, Université de Montpellier, 34095 Montpellier Cedex 5, France; ^2^Institut Européen des Membranes, IEM UMR-5635, Université de Montpellier, ENSCM, CNRS, Place Eugène Bataillon, 34095 Montpellier Cedex 5, France; ^3^Hydro-Québec's Research Institute, Varennes, QC, Canada J3X 1S1; ^4^Centre de Technologie de Montpellier, Université de Montpellier, 34095 Montpellier Cedex 5, France

## Abstract

Nanocomposites physical properties unexplainable by general mixture laws are usually supposed to be related to interphases, highly present at the nanoscale. The intrinsic dielectric constant of the interphase and its volume need to be considered in the prediction of the effective permittivity of nanodielectrics, for example. The electrostatic force microscope (EFM) constitutes a promising technique to probe interphases locally. This work reports theoretical finite-elements simulations and experimental measurements to interpret EFM signals in front of nanocomposites with the aim of detecting and characterizing interphases. According to simulations, we designed and synthesized appropriate samples to verify experimentally the ability of EFM to characterize a nanoshell covering nanoparticles, for different shell thicknesses. This type of samples constitutes a simplified electrostatic model of a nanodielectric. Experiments were conducted using either DC or AC-EFM polarization, with force gradient detection method. A comparison between our numerical model and experimental results was performed in order to validate our predictions for general EFM-interphase interactions.

## 1. Introduction

Interphases in polymeric nanocomposites have long been considered responsible for the properties changes of these materials, enhancing their mechanical and thermal performances as well as their dielectric ones [1]. This interphase is considered to be a region surrounding the surface of the particle that has different properties than initial components, particle, and matrix. Among these properties, the dielectric permittivity of this region is supposed to be either higher or lower than both matrix and filler at low nanofiller concentration due to rearrangements of polymer chains [2, 3]. The unique permittivity of the interphase explains the behavior of this property of nanocomposites at the macroscopic level, which does not follow conventional mixture laws. Hence, there is a great interest to consider the interphase locally and measure its intrinsic properties such as its dielectric constant and dimensions.

This region possesses nanometric dimensions, which leads to a need for a nanoscopic characterization method to probe it. Among all current nanoscopic techniques [4, 5], atomic force microscopy (AFM) constitutes a highly versatile one that can be adapted through different modes to the investigation of numerous properties [6].

AFM nanomechanical modes have been commonly used for materials that show at interphase regions mechanical properties different than matrix and filler [7–10]. In the same perspective, authors in [9, 10] used electrostatic force microscopy (EFM) to perform dielectric spectroscopy at the nanoscale. They studied the interphase dynamic properties by comparing pure polymer regions to particle filled polymer regions. The EFM is part of the AFM family which uses a conductive tip and a metallic substrate. The system is either DC or AC electrically polarized, which allows the detection of electrostatic forces or force gradients. One more recent EFM study found a different EFM apparent filler diameter depending on whether the filler is embedded, or not, into a polymeric matrix [11]. The authors attributed those changes to the presence of an interphase surrounding filler particles.

Since the dielectric permittivity can be used as the fingerprint of dielectrics, EFM is widely used to study dielectric materials at a nanometric scale [12, 13]. However, in order to detect the interphase with EFM, one should be able to detect the particle and interphase association when totally embedded in the matrix, as well as be able to differentiate between the presence and absence of interphase. Therefore, it is necessary to expose the problem of the detection or distinction of superposed dielectric layers that possess nanometric 3D-dimensions.

Recently, some authors have determined the composition of the core of viruses or bacteria based on the change of the measured effective permittivity of the core-shell like structure [14, 15]. They used an AC voltage and an additional lock-in amplifier in order to extract the 2*ω* component of the electrostatic force.

Furthermore, although few studies where the presence of the interphase in nanodielectrics has been investigated with EFM can be found in the literature [11], an attempt at theoretical interpretation has not been proposed yet [16]. A specific fundamental study of such systems is needed in order to accurately analyze EFM measurements performed on a nanodielectric and then to conclude whether the observed changes should be associated with the interphase or to nonlocal contributions.

In this work, we first report numerical simulations of the EFM signal response to a nanodielectric sample, in order to understand how to attest to the presence or the absence of the interphase with this technique and discuss its benefits and limitations.

In the second time, according to simulations and the current state of the art of nanodielectrics, samples are designed and synthesized to model a part of a nanodielectric material where particle and interphase are materials of known permittivity, geometry, and dimensions.

The set of model samples presented in this work is used to assess experimentally the sensitivity of EFM to the thickness of the artificial interphase. Experimental signals interpretation is also inspired by the general simulations over a nanodielectric. During these measurements, two EFM techniques are employed, different from those mentioned in [14, 15], to detect the shell of our nanoparticles. We use the DC-biased force gradient detection method (available on most microscopes) in addition to the AC-force gradient detection method. During AC measurements, the 2*ω* component is extracted with an external lock-in amplifier.

Finally, we adapt our Comsol® numerical force model to force gradient calculations over our samples. Previous reported analytical force models were only applicable to samples with lateral homogeneity higher than EFM resolution [17]. Moreover, previous numerical force gradient models only treated the case of one-layered dielectric films [18]. In [14, 15], other authors successfully used Comsol simulations to correlate measurements over nanoparticles and core-shell particles in the AC-force detection mode. A similar model was used in [19] to get information about the depth of carbon nanotubes in a matrix, also measured using the AC-force detection mode. In our work, the correlation of experiments to simulations is performed in order to conclude on the adaptability of our model for both force and force gradient calculations and for the interpretation and the quantification of the EFM signal with nanocomposites.

Materials, simulations, and experiments are presented in Section 2. Simulations and EFM measurements are then described and discussed in Section 3. EFM experiments and simulations are compared in Section 4, followed by the conclusions of this work in Section 5.

## 2. Samples Description

The materials specifically designed and fabricated for this study are based on spherical polystyrene (PS) particles deposited on a metallic substrate and covered by an alumina (Al_2_O_3_) shell of different thicknesses. PS particles have a 230 nm approximate diameter and alumina shells a thickness of 20, 60, 100, and 200 nm.

### 2.1. PS Deposition

PS particles of 1 *μ*m initial diameter (Sigma-Aldrich, ref: 89904) were deposited on substrates using the self-assembly property of PS spheres (PSS) [21–23].

**Figure 1:**
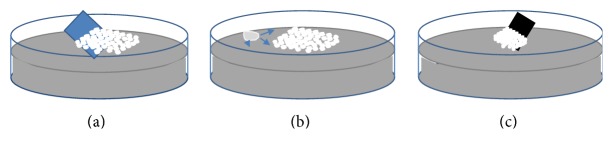
PSS deposition process: (a) insertion of glass substrates with the solution of organized PSS on the water surface; (b) addition of SDS drop; (c) transfer on substrates of PSS monolayer.

We dropped 40 *μ*L of the solution diluted with equal amount of ethanol, on a glass substrate of 2*∗*2 cm^2^ approx. Glass substrates were treated with oxygen plasma for 2 minutes in order to increase the hydrophilicity of the surface. During deposition, we fixed the substrate at 45° approx. from the horizontal side. Then, we gently immersed the glass substrate into water as shown in Figure 1(a) [23]. We used a glass vessel of 7 cm diameter filled with 80 mL of Milli-Q water. PSS start to organize themselves on the water surface forming a hexagonally close-packed monolayer. We add a drop of sodium dodecyl sulfate (SDS) solution (10 wt% in water) on the water surface to consolidate the particles (Figure 1(b)). Then, we transfer PSS monolayer on the substrates prepared for EFM characterization (Figure 1(c)). Silicon substrates with a thin native oxide layer have been used with 50 nm sputtered gold above a 10 nm chrome fixing layer to render the surface conductive. Before PSS deposition, EFM substrates were also kept for 2 minutes in oxygen plasma.

### 2.2. PSS Diameter Modification: Plasma Etching

The films of PS particles were etched in a plasma reactor with oxygen. The samples were inserted into the reactor chamber under vacuum (~0.011 mbar). Oxygen was introduced using a needle valve, and the pressure was equilibrated to 0.6 mbar by adjusting the valve. After equilibrium pressure is reached, a radio frequency power of 50 W at 0.15 A has been applied until obtaining the desired diameter (around 230 nm after 15 min etching, measured with AFM) [24].

### 2.3. Alumina Shell Deposition by ALD

**Figure 2:**
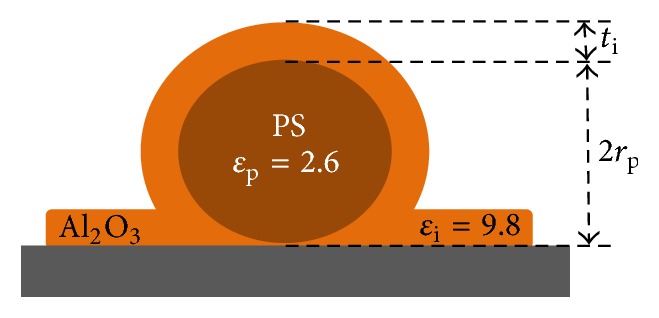
Studied samples: polystyrene nanoparticles (PS) with alumina layer (Al_2_O_3_).

The atomic layer deposition (ALD) method was used in order to grow the different alumina layers above nanoparticles (see [25, 26] for further explanations). The final configuration of the samples is presented in Figure 2.

ALD is a thin film deposition method. The thickness of the film is precisely controlled at the atomic level. The deposition is based on a sequential chemical reaction between gas precursors and the surface of the material. After each cycle of one precursor, an inert gas is introduced to remove the remaining precursor and the resulting by-products.

For alumina deposition, we used trimethylaluminium, (TMA) or (Al(CH_3_)_3_), as an aluminum precursor, H_2_O as an oxygen source, and argon as an inert gas. The generally accepted and dominant reaction mechanism occurring at steady state [27] is

(1) _OH+AlCH33g⟶_O_Al_CH32+CH4g

(2) _CH3+H2Og⟶_OH+CH4g

A custom-made ALD reactor was used for the synthesis of Al_2_O_3_ films [28]. ALD was performed using sequential exposures of TMA and H_2_O separated by a purge of argon with a flow rate of 100 standard cubic centimeters per minute (sccm). The deposition regime for Al_2_O_3_ consisted of 0.1 s pulse of TMA, 40 s of exposure, and 60 s of purge with argon followed by 2 s pulse of H_2_O, 40 s of exposure, and finally 60 s purge with argon. The thickness of the film was determined by the number of ALD reaction cycles. Thus, 20, 60, 100, and 200 nm thickness Al_2_O_3_ layers were deposited by 100, 300, 500, and 1000 numbers of ALD cycles, respectively. The deposition was performed at 80°C. The typical growth rate for Al_2_O_3_ coating during these cycles is found to be 0.2 nm per cycle. The thickness error is between 5 and 10% determined after the characterization of reference samples with ellipsometry and profilometry.

## 3. Experimental Methods

### 3.1. Physics Governing EFM Tip-to-Sample Interaction

The interaction between an EFM tip and an insulator is a combination of a capacitive force between induced charges on electrodes due to the capacitance *C* of the probed region, and a columbic force between local surface charges *q*_*s*_ (if present) and their image charges on the tip −*q*_*s*_ [29, 30]. The total tip-sample voltage is due to externally applied voltages, DC and/or AC voltages, as well as those due to the existing tip-to-sample work function difference (well known as surface or contact potential difference *V*_CP_), to which other externally induced voltages can be added like those resulting from polarization, illumination, mechanical stress, and so forth.

The general equation of the force *F* that describes these interactions is defined as follows:

(3) $$\begin{array}{c} F=\frac{1}{2}\frac{\partial C}{\partial z}\Delta V^{2}+\frac{q_{s}q_{t}}{4\pi \varepsilon_{0}z^{2}}. \end{array}$$

*z* is the instantaneous distance between the tip apex and the sample surface, *q*_*t*_ is the sum of all charges interacting with the surface static charges *q*_*s*_, and the total voltage difference Δ*V* is expressed as

(4) $$\begin{array}{c} \Delta V=V_{\text{DC}}+V_{\mathrm{AC}}\sin\left(\;\omega t\;\right)+V_{\text{CP}}. \end{array}$$

*V*_DC_ and *V*_AC⁡_sin⁡*ωt* are the DC and AC externally applied voltages, respectively. *V*_CP_ can be measured by Kelvin force microscopy [31]. In the case of a dielectric sample, it mostly corresponds to the work function between the tip and the metallic substrate [18]. Note that other external voltages mentioned before are neglected or can be included within *V*_CP_.

*q*_*t*_ is expressed as follows:

(5) $$\begin{array}{c} q_{t}=q_{s}+q_{\text{DC}}+q_{\mathrm{AC}}+q_{\text{CP}}, \end{array}$$

where *q*_DC_ = *CV*_DC_, *q*_AC⁡_ = *CV*_AC⁡_sin⁡*ωt*, and *q*_CP_ = *CV*_CP_ are the capacitive charges due to *V*_DC_, *V*_AC⁡_sin⁡*ωt*, and *V*_CP_, respectively.

The force acting on the tip becomes equal to

(6) F=qsqs+CVDC+CVAC⁡sin⁡ωt+CVCP4πε0z2+12C′VDC+VAC⁡sin⁡ωt+VCP2.

The development of this expression shows that it can be expressed as the sum of DC, *ω*, and 2*ω* components:

(7) FDC=12C′VDC2+Cqs4πε0z2+C′VCPVDC+14C′VAC⁡2+qs24πε0z2+CqsVCP4πε0z2+12C′VCP2,Fω=qs4πε0Cz2+C′VDC+VCPVAC⁡sin⁡ωt,F2ω=−14C′VAC⁡2cos⁡2ωt.

At this stage, one should note that measurements under DC or AC excitation modes can be carried out by either force detection or force gradient detection. However, the force gradient detection method is expected to offer higher lateral resolution [32].

Hence, the force gradient *G* = ∂*F*/∂*z* is deduced from previous expressions and becomes similar to the force; the sum of three components is

(8) GDC=12C′′VDC2+qs4πε0C′z2−2Czz4+C′′VCPVDC+12C′′VCP2+qs4πε0C′z2−2Czz4VCP+14C′′VAC⁡2−2qs24πε0z3,Gω=qs4πε0C′z2−2Czz4VAC⁡+C′′VDC+VCPVAC⁡·sin⁡ωt,G2ω=−14C′′VAC⁡2cos⁡2ωt.

The extraction of the 2*ω* component of force or force gradient is possible with a lock-in amplifier which suppresses all the noises and electrical responses that are not at the electrical double frequency and which are not only dependent on the capacitance of the probed region [19].

### 3.2. Detection Protocols

EFM measurements were performed in air with a commercial AFM (Bruker, previously Veeco, Enviroscope™). The probe consists of metal covered tips (Budget Sensors: ElectriMulti75-G and *μ*masch: HQ:NSC18/Pt) supported by a cantilever electrically connected to a metallic sample holder and biased at an electrical potential.

We used DC-biased amplitude modulation EFM (AM-EFM) and AC-biased AM-EFM in a double-pass configuration [29]. The probe is excited at its first eigenmode by a piezoelectric bimorph actuator. During the first scan, tapping mode is used to extract the sample topography. At the second scan, the sensor is lifted by a known distance from the surface and controlled to follow the topography profile acquired from the first scan. During this scan, a voltage difference is applied between the probe and the sample holder and the mechanical oscillation amplitude is reduced by a factor 3 to stay in the linear regime. The acting electrostatic force gradients reduce the effective spring constant of the probe and, consequently, modify its resonance frequency. Experimentally, the resonance frequency shifts Δ*f* are extracted during the second scan by maintaining the phase shift constant through the modification of the exciting frequency.

In the linear regime, Δ*f* and electrostatic force gradients, *G* (N m^−1^), are related by the following equation:

(9) $$\begin{array}{c} \Delta f≅-\left(\;\frac{f_{0}}{2K}\;\right)\times G, \end{array}$$

where *K* is the stiffness of the cantilever and *f*_0_ its free resonance frequency. The expansion of the expression of the DC-force gradient as explained in the above section shows that Δ*f*(*V*_DC_) curve is a second-order polynomial response:

(10) $$\begin{array}{c} \Delta f≅\alpha {V_{\text{DC}}}^{2}+\beta V_{\text{DC}}+\gamma , \end{array}$$

where *α* = −(*f*_0_/4*K*) × *C*′′, with *C*′′ being the second derivative of the probe-to-sample capacitance. From *α* expression, it can be deduced that this coefficient mainly depends on the dielectric properties of the probed region; while for *β* and *γ*, in addition to the capacitance derivatives, these coefficients also depend on local surface charges and contact potential.

For DC measurements, EFM maps were performed at different regions of all samples at *V*_DC_ = 0, 5, and −5 V. For each voltage, we extracted at the top of the spherical particles the average Δ*f* on few pixels. At several tip-sample distances, Δ*f*(*V*_DC_) curves have been fitted with a polynomial function similar to (10) in order to extract the corresponding *α* coefficient. This coefficient has been used in [13], associated with modeling with the Equivalent Charge Method, with the aim of extracting the dielectric permittivity of polyvinyl acetate particles placed into a polystyrene matrix.

For AC measurements, *G*_2*ω*_ values have been provided by the use of an external lock-in amplifier where the feedback loop controls the frequency shift keeping the phase shift constant. The AC electrical excitation frequency *ω* is chosen much lower than the resonance frequency of the cantilever to avoid interference.

Although *G*_2*ω*_ accounts for the unique capacitance contribution of the system, DC-frequency shifts are important in the way that they are usually accessible in any standard AFM equipped with EFM module. AC measurements are usually more complicated to extract. In our case, we used a signal access module with an external lock-in amplifier and an external frequency generator in order to demodulate the 2*ω* component from the signal at the second pass.

### 3.3. Modeling Description

To model the force acting on the tip over the sample, we used the AC/DC module (Electrostatics) of Comsol Multiphysics software. This software uses finite-element method to solve Poisson's equation for the tip/sample system, which results in a map of the electrostatic potential distribution. The Maxwell stress tensor is then calculated and its integration around the tip surface gives the resultant electrostatic force at each position of the scan line [33].

**Figure 3:**
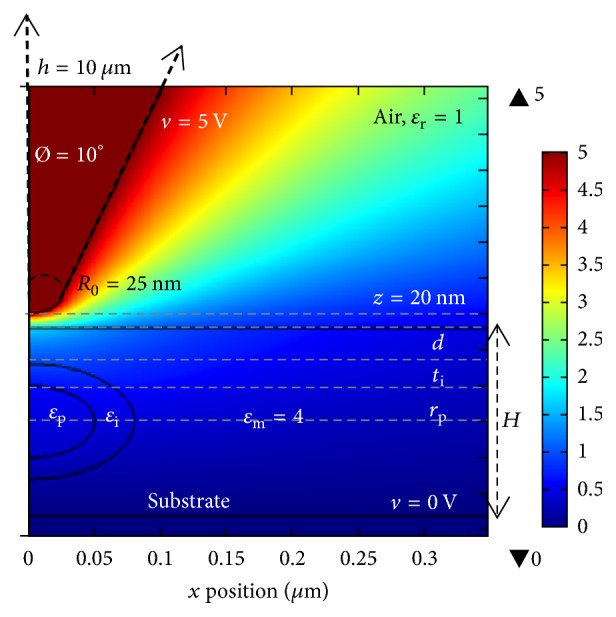
Electric potential map in volts obtained with a 2D axisymmetric model of the EFM tip, nanodielectric sample, and substrate.

The probe was modeled as presented in Figure 3. The probed region of the nanodielectric model consists of a nanoparticle surrounded by an interphase placed in a matrix. The polymer matrix is represented as a disk of 20 *μ*m diameter, thickness, *H*, and dielectric constant, *ε*_m_. The nanoparticle is modeled as a solid sphere of radius *r*_p_ and dielectric constant *ε*_p_. The particle is buried at a certain depth *d*, from the matrix upper and lower surfaces. The interphase around the nanoparticle was modeled as a spherical shell of thickness *t*_i_ and dielectric constant *ε*_i_. A first presentation of this model can be found in [16].

When measuring the force at the top of particle/interphase assembly, our system is axisymmetrical. Moreover, when the tip is placed over the matrix alone, relatively far from the particle, the tip is not influenced by the particle. The system is thus similar to an EFM tip over the matrix without the inclusion. In this case, our measurements have been performed in axisymmetric dimensions as well. However, for the calculations of the force on a scan line 2D modeling has been used. The probe was biased at 5 V whereas the substrate was grounded. We studied the *z* component of the electrostatic force similarly to previous finite-element EFM models [34].

## 4. Results and Analysis

### 4.1. Numerical Simulations

#### 4.1.1. EFM Signal over a Nanodielectric: Particle-Interphase-Matrix

**Figure 4:**
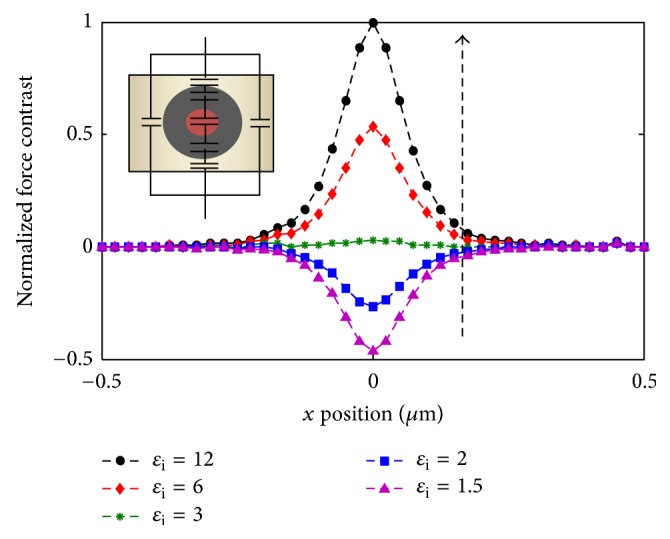
EFM normalized force contrast on a 1 *μ*m scan over a nanodielectric of *ε*_m_ = 4, *ε*_p_ = 10, *r*_p_ = 25 nm, and *t*_i_ = 20 nm and at 15 nm from upper and lower surfaces; inset: capacitance model of the nanodielectric comparing center to border scan line regions.

We present in Figure 4 the result of 2D axisymmetric calculation of the force contrast normalized to the maximum contrast among the curves (*ɛ*_i_ = 12), on a scan line over the nanodielectric probed region for different interphase permittivities. These curves show that although the probe encounters three main regions of different capacitances, the expected signal is characterized by a single maximum with a certain half-width at half maximum (HWHM). The force contrast and HWHM depend on the interphase permittivity and thickness; both can either increase or decrease and the contrast can even change in sign. We explain this behavior by the fact that EFM detects the particle and interphase assembly as one apparent particle with one apparent effective permittivity, while having a radius *D* = *r*_p_ + *t*_i_ [15]. Thus, if our system is assimilated to an association of capacitors in series in the *z* direction (inset of Figure 4), the apparent permittivity might become higher or lower than that of the matrix depending on the relative material permittivities and dimensions, which explains contrast variations. This result shows that the presence and the characteristics of the interphase can be proved by studying either signal intensity, contrast, or HWHM.

Moreover, it can be noticed from Figure 4 that for certain critical interphase permittivities, *ɛ*_ic_, and thicknesses, *t*_ic_, the contrast can greatly decrease and reach the detectability limit of EFM. This indicates that certain conditions will not allow detecting the interphase.

#### 4.1.2. Interphase Detectability Limits

*(i) No Matrix Layer above and below the Particle*. In order to detect the interphase, two main conditions should be accomplished. The first one is to detect the particle and interphase assembly when totally embedded within the matrix, and the second one is to detect a difference between the presence and absence of interphase.

Firstly, to accomplish the first condition, the force contrast between the matrix and particle/interphase assembly must be detectable. Thus, the contrast Δ*F* should be higher than the noise of 10 pN for soft cantilevers, typically [35]. Note that force gradient detection generally shows higher sensitivities than force detection. However, the limit on interphase conditions obtained with the coming simulations of the force still holds for force gradients as an upper extreme limit. Force simulations are also useful for most users of electrical scanning microscopies that often opt to measure electrostatic forces, rather than force gradients [14, 15, 36, 37].

**Figure 5:**
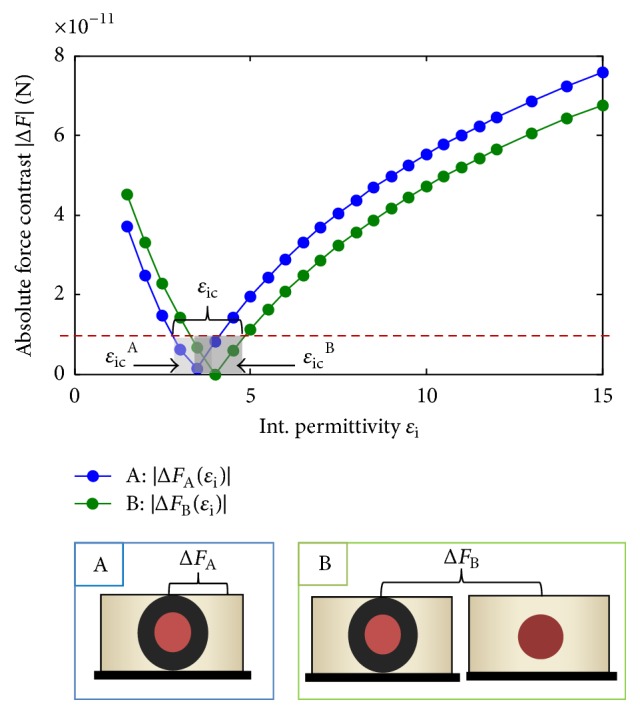
Force contrast absolute values between particle-interphase assembly and matrix, for a nanoparticle of *ɛ*_p_ = 10, a 25 nm radius, and 20 nm interphase thickness; inset A: particle-interphase assembly detection, inset B: interphase detection (assembly compared to particle alone).

As an example, we present the case of alumina nanofillers with a permittivity *ε*_p_ = 10 and a radius *r*_p_ = 25 and 50 nm placed within a matrix of permittivity *ɛ*_m_ = 4. Trace A of Figure 5 shows the variation of the absolute values of the force contrast versus interphase permittivity for interphase thickness *t*_i_ = 20 nm (inset A). We thus deduce the interval of critical permittivities *ɛ*_ic_^A^ for which the assembly is undetectable with these values of *r*_p_ and *t*_i_. Note that in the case of samples where fillers are not totally embedded in the matrix, producing a bulge, this first condition can be ignored and the coming one becomes sufficient.

Secondly, to accomplish the second condition, we present on the green curve of Figure 5 the variation of the force difference between the presence and absence of interphase (inset B). This difference must also exceed the detectability limit, which is verified for interphase permittivities outside the interval *ɛ*_ic_^B^.

**Figure 6:**
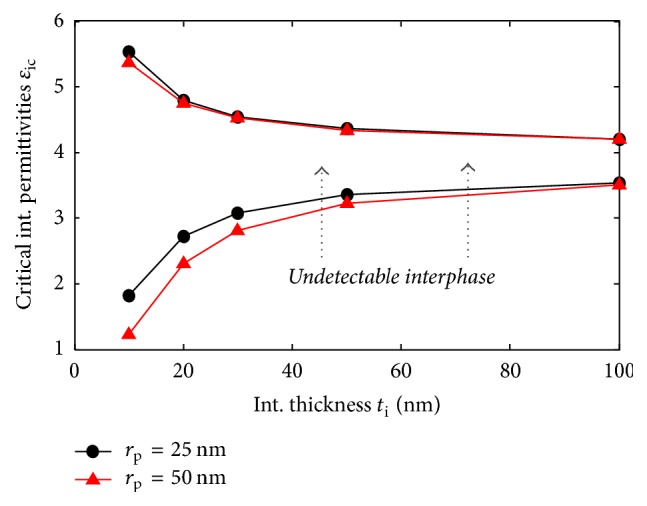
Final critical combination of *ε*_i_ values, [*ε*_iMin_, *ε*_iMax_], at different *t*_i_ for interphase detection of a nanoparticle of *ε*_p_ = 10 having a 25 nm and 50 nm radii in a matrix of *ε*_m_ = 4 at zero depth.

**Figure 7:**
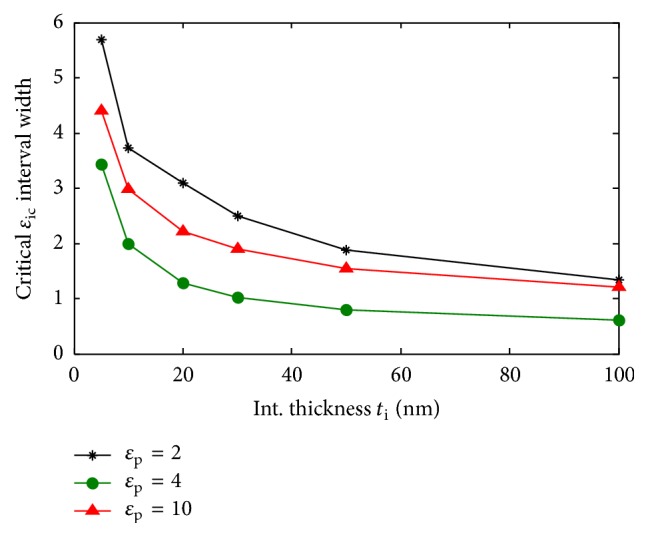
Width of critical interphase intervals for a particle of 50 nm radii for different particle permittivities below (*ɛ*_p_ = 2), equal to (*ɛ*_p_ = 4), and higher than matrix permittivity.

The final right condition is the combination of these two intervals, which goes in this case, from the minimum of the first one to the maximum of the second one. We present in Figure 6 the obtained critical interphase permittivities at different interphase thicknesses for a particle of *ɛ*_p_ = 10 and two radii, *r*_p_ = 25 and 50 nm. It can be clearly noticed that the width of these critical intervals decreases when the interphase thickness increases. In Figure 7, we present the variation of the width of the critical interphase permittivities intervals corresponding to a particle of 50 nm radius, for *ɛ*_p_ = 2, 4, and 10, that model, respectively, a particle of permittivity, lower, equal, and higher than the matrix. The width decreases when the particle and matrix permittivities are equal. This result appears logical, since, in such cases, any perceived contrast must be due to the interphase. Thus, higher interphase thicknesses and closer particle and matrix permittivities seem to improve the probability of interphase detection.

**Figure 8:**
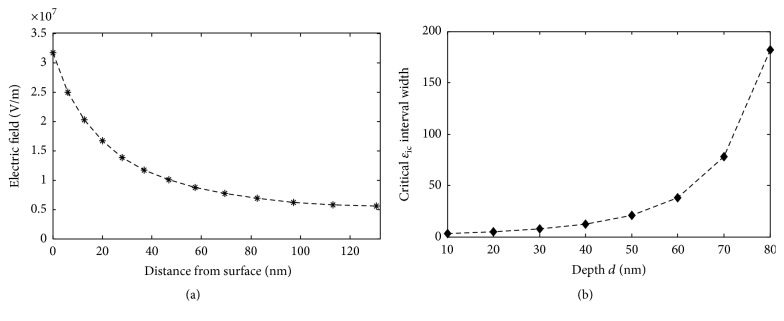
(a) Electric field intensity into a dielectric sample of 100 nm thickness and a relative permittivity of 4; (b) width of the critical interphase permittivities interval for a particle of 50 nm radius, *ε*_p_ = 10 and 20 nm interphase thickness at different depths from upper and lower surfaces.

*(ii) Matrix Layer above and below the Particle*. In real practical cases, when preparing thin slices for EFM studies, a thin layer of the unchanged polymer matrix is highly probable to exist over and below the particle due to a nonperfectly controlled cutting process of the bulk material. As previously reported in [16], the presence of a matrix layer decreases the contrast. This signal change can be explained by the fact that the intensity of an electric field in an EFM configuration highly decreases as it penetrates the material (Figure 8(a)) [18]. Moreover, the matrix layer in the central region balances the central effective permittivity with that of the matrix at the borders. Thus, an additional matrix layer is supposed to increase undetectable interphase permittivities *ɛ*_ic_ intervals.

We study in Figure 8(b) the width of *ɛ*_ic_ intervals for a particle of *r*_p_ = 25 nm and *ε*_p_ = 10. A fast increase of *ɛ*_ic_ interval width can be noted as expected due to the decrease of the electrical field when it penetrates the sample (Figure 8(a)) in addition to center and borders permittivities equalization. The matrix layer thickness above particle/interphase assembly is hence found to decrease interphase detection probability.

Note that the adopted method for extracting interphase critical properties stays valid for any other value of sensitivity limit.

*(iii) Use of Simulation for Samples Design*. We used the graph presented in Figure 6 in order to design the nanodielectric model samples to synthesize. These model samples were used to verify experimentally, in the following paragraph, the expected behavior of EFM simulations presented in the previous section and to help us set and calibrate the protocol for interphase investigation. Then, we selected the samples described in Section 2 for whose interphase variations must be detectable with EFM. We used samples of PS spherical particles (*ɛ*_p_ = 2.6, *r*_p_ = 125 nm) covered by a controlled Al_2_O_3_ interphase (*ɛ*_i_ = 9.8, *t*_i_ = 20, 60, 100, 200 nm). Theses samples meet our criteria for the following reasons:

1. A higher particle diameter seems to decrease the critical interphase interval upper limit *ɛ*_ic_^B^. Hence, in our case of polystyrene particle, for all interphase thicknesses, an aluminum oxide shell of 9.8 dielectric permittivity lies within the detectable region.
2. Although, in nanodielectrics, the interphase dielectric permittivity variation compared to bulk polymer is not expected to be too high, water absorption at the interfacial particle region is commonly reported [38, 39]. Water molecules within the interphase increase its effective dielectric constant justifying the possibility of a high dielectric interphase permittivity. Additionally interphases thicknesses in literature have been reported to vary between few nanometers [11, 40], up to 200 nm [41].
3. Alumina layer thickness to particle diameter ratios lie within the range of simulated values. Moreover, they are in agreement with the thicknesses reported in the literature.

It must be noticed that our system particle-shell can be introduced into a matrix to represent a complete nanodielectric model. However, in this work, we investigate first the way to detect the shell before being covered, which electrostatically represents, similarly to a nanodielectric, a multilayered system with 3D finite-size.

### 4.2. EFM Characterization

An earlier version of the DC measurements results of Section 4.2 is presented in [20].

Hereafter, we present EFM force gradient detection measurements in both DC and AC polarization to detect alumina shells covering PS nanoparticles. The adaptability of DC-frequency shift detection compared to AC measurements constitutes a versatile method to investigate dielectric properties using standard electrostatic force microscopes without external connections.

**Figure 9:**
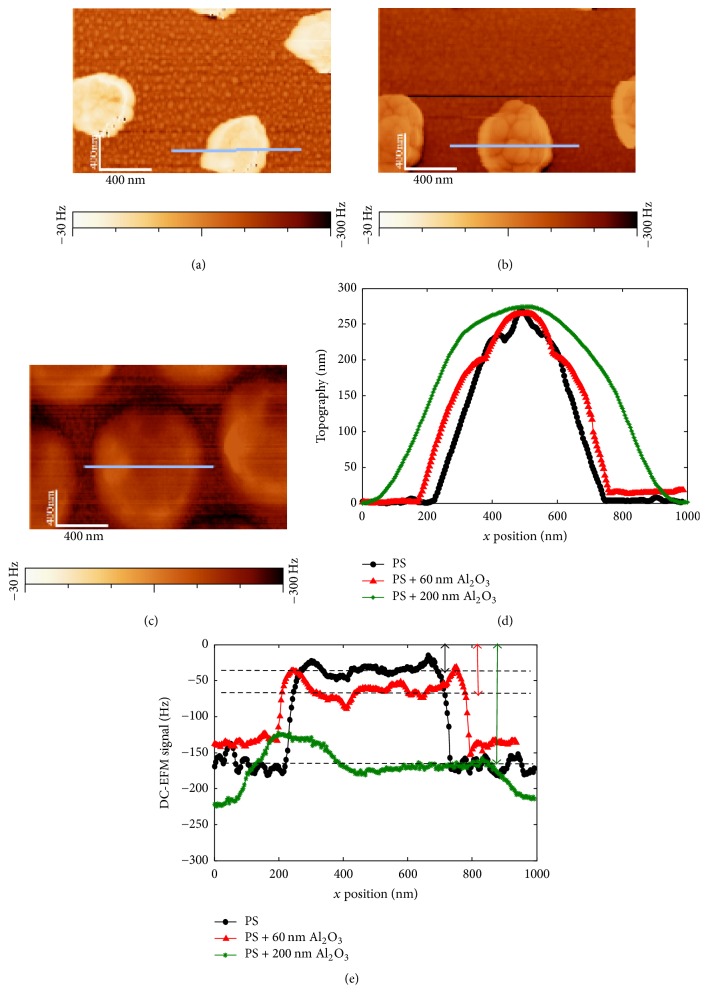
(a), (b), and (c) EFM *G*_DC_ detection electrical signal for PS and PS with 60 nm and 200 nm Al_2_O_3_, respectively; (d) Cross-sectional topography profiles and (e) EFM signal for studied PS particles at *V*_DC_ = 5 V and *z* = 26 nm. Reprinted with permission from [20]. Copyright IEEE 2016.

**Figure 10:**
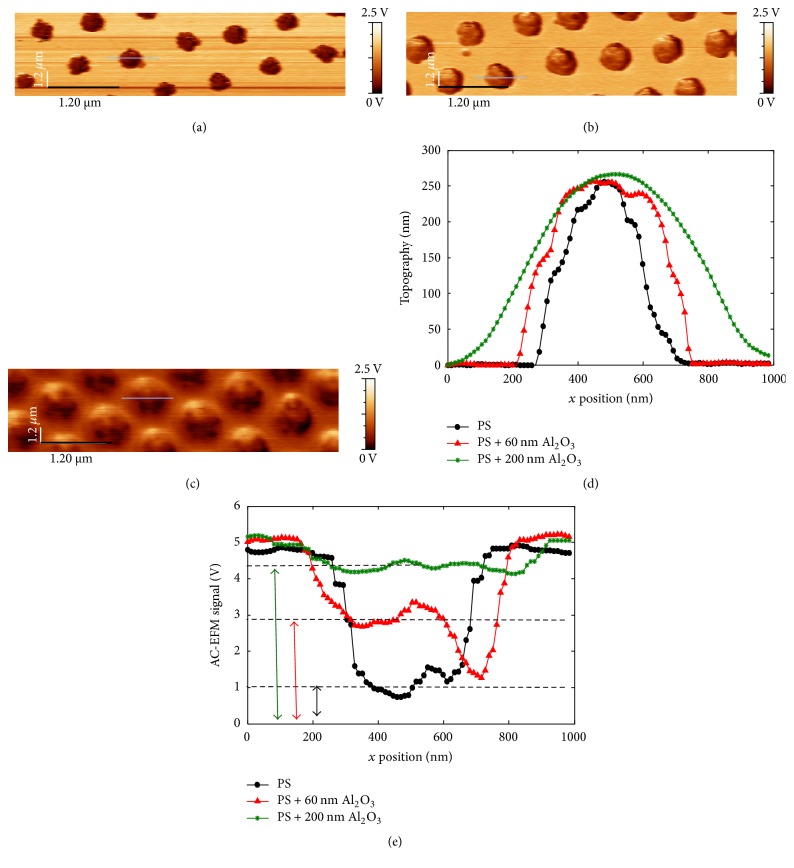
(a), (b), and (c) EFM *G*_AC⁡_ (2*ω*) detection electrical signal for PS and PS with 60 nm and 200 nm Al_2_O_3_, respectively; (d) Cross-sectional topography profiles and (e) EFM signal for studied PS particles at *V*_AC⁡_ = 3.5 V and *z* = 30 nm.

Figures 9 and 10 show topography and EFM images of PS nanoparticles, without coating (a) and with 60 nm (b) and 200 nm alumina coating (c). Cross-sectional profiles along the main axis of the particles, presented in topography images of Figures 9(d) and 10(d), show similar height, around 250 nm, for the compared particles. This suggests that the diameter of these particles is of the same order since alumina is supposed to equally cover the whole sample (see Figure 2). Note that height profiles are exempt of Gaussian effect, due to the tip radius, compared to lateral profiles. However, the difference in lateral profiles is consistent with the thickness of the added alumina layers (Figure 9). EFM images of Figures 9(e) and 10(e) show raw EFM signals between differently covered PS particles in *G*_dc_ and *G*_2*ω*_ detection modes, respectively.

For PS nanoparticles without shell, EFM contrasts result from the difference of the detected force gradient between the nanoparticle and the metallic substrate. Concerning covered PS particles, the signal difference originates from the difference between the covered particle with the alumina film alone covering the metallic substrate. However, contrasts obtained by the double-pass method, or by retracing sample topography, have been found to be highly influenced by tip-substrate distance, or interelectrodes distance change as the tip is scanning the surface. Van Der Hofstadt et al. [37] called this effect as topography cross-talk, first introduced by [14]. In our case, the bottom of our samples, or the regions between particles, does not possess the same composition but varies with each shell thickness. The knowledge of the thickness of the sample at each position is not straightforward with topography measurements. The method proposed in ref [37] cannot be adopted here. Hence, to counteract cross-talk effects, instead of the comparison of EFM image contrasts, we compare the raw signal for similar lift heights, at the center of particles. We find that the signal intensity increases with the presence of an alumina shell and increases with increasing alumina thickness for particles of comparable diameters (around 250 nm) (Figures 9 and 10). The change in the DC and 2*ω* signal absolute values at particles center and for same lift distances shows the sensitivity of this method of signal analysis to the presence of the shell.

As explained in Section 3.1, DC measurements are sensitive to the sample surface potential. Hence, in addition to the raw signal, and for further accuracy, we studied for several particles the behavior of the purely capacitive *α* parabolic coefficient parameter, extracted over the central region of particles, as explained in Section 3.2.

**Figure 11:**
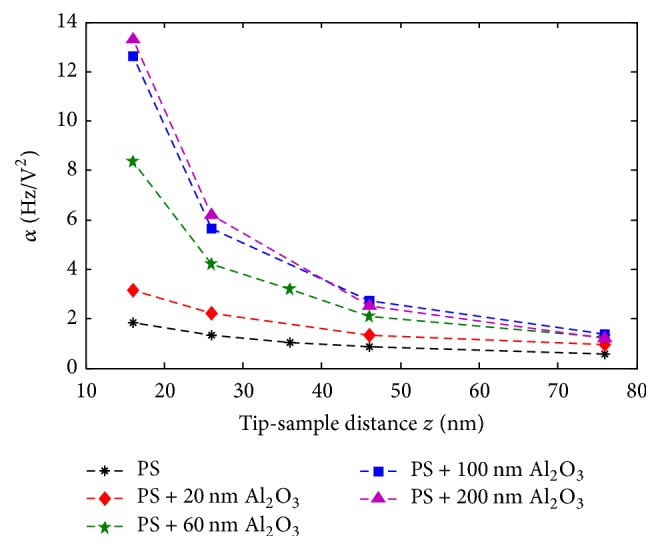
*α* coefficient versus tip-sample distance for PS nanoparticles with and without alumina shell. Reprinted with permission from [20]. Copyright IEEE 2016.

Figure 11 represents curves of the experimental *α* parameter versus tip-sample distance *z* for PS samples covered by different Al_2_O_3_ shell thicknesses. Firstly, it can be noticed that *α* decreases with increasing tip-sample distance. Decreasing *α* values are explained by their proportional dependance to the electrostatic force gradient that itself decreases with *z*. Hence, it can be deduced that measurements at short distances (≲26 nm) are more sensitive to small variations between samples and are consequently considered to be more accurate. Secondly, for the same tip-sample distance, PS nanoparticles with an alumina shell exhibit clearly higher *α* values than uncoated PS spheres, whereas *α* increases with the thickness of the shell. One must note that when the tip probes the bottom regions of the sample, the surface of interaction with the surroundings increases. The signal can be influenced by nonlocal contributions. Consequently, a precise measurement is mostly attributed to the central regions of the particles.

**Figure 12:**
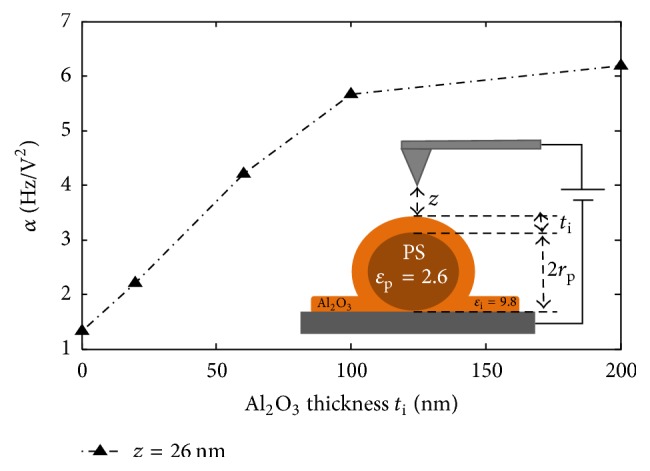
*α* coefficient for different alumina shell thicknesses over PS nanoparticles measured at a constant tip-sample distance of 26 nm. Reprinted with permission from [20]. Copyright IEEE 2016.

The difference between the presence and absence of the shell shows the good sensitivity of the DC-*α* coefficient extraction to detect the presence of a dielectric layer over PS nanoparticles, without the effect of surface potential. Moreover, the significant increase of *α* with the alumina shell thickness shows that the DC-force gradient detection method is well adapted to evaluate the thickness of a dielectric alumina layer above nanospheres of polystyrene of 250 nm diameter approx. Detectable thicknesses in our case are comprised in a range between 20 nm and 200 nm. In particular, we show in Figure 12 the trend observed for *α* variation versus alumina shell thickness at a constant tip-sample distance. We note that the slope of the curve is high up to 100 nm and becomes weaker beyond this value. Thereby, it can be deduced that the sensitivity to alumina thickness is limited to a certain range of thicknesses.

**Figure 13:**
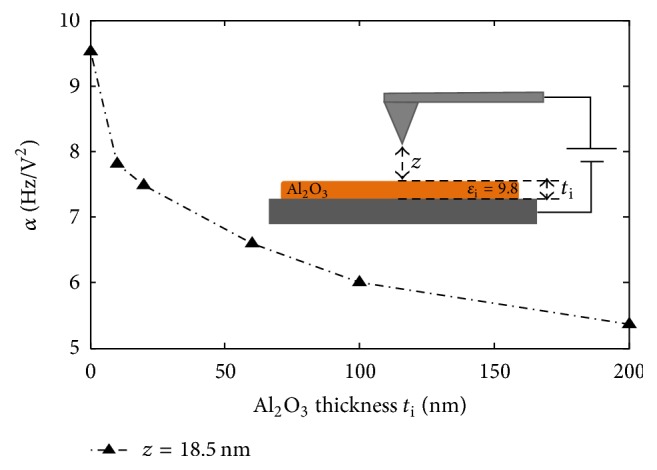
*α* coefficient for different alumina thicknesses covering bare metallic substrates for a constant tip-sample distance of 18.5 nm. Reprinted with permission from [20]. Copyright IEEE 2016.

*(i) Evidence of a Dielectric Effect of Alumina Coating*. Although shell addition induces remarkable morphological changes, we will prove that the effects observed in previous sections are mainly due to the dielectric properties of the coating. In fact, the increasing signal cannot be caused by the increased distance between electrodes induced by thicker layers: from Figure 11, as we compare *α* between PS at *z* = 76 nm and PS + 60 nm Al_2_O_3_ at *z* = 16 nm, where both interelectrode distances are equal to 326 nm, the same tendency is conserved. Additionally, higher separation instances between electrodes are physically supposed to decrease the signal. Moreover, force gradient changes cannot be the result of the increasing thickness of PS + Al_2_O_3_ probed region: Figure 13 shows that the increase of a homogenous dielectric film thickness decreases the signal. Consequently, we explain the enhanced EFM signal by the increase of the resulting effective dielectric constant of covered particles compared to bare particles or particles with lower additional layer thickness. Therefore, shell permittivity must be higher than particle one. In our case, this is well verified with an alumina layer, for which the relative permittivity is equal to 9.8, which is higher than PS permittivity that is equal to 2.6. Furthermore, alumina dielectric polarization effect on the global capacitance of the probed region becomes weaker after certain thicknesses that approach the effective permittivity to that of the shell. Beyond this thickness, the tip interacts with the multilayered material as a homogeneous one, as alumina alone. This explains the saturation-like values of *α* versus Al_2_O_3_ height.

### 4.3. Simulations versus Experiments

In this section, a qualitative correlation between experiments and electrostatic force simulations presented during the beginning of the paper is first reported. Next, an attempt to quantify EFM signals in DC measurements is addressed by following specific calibration steps and adapting our Comsol model to the exact geometry and nature of the samples, as well as the measurement of force gradients instead of forces.

#### 4.3.1. EFM Signal and Particle-Interphase Assembly Detection

We showed in Figures 9(e) and 10(e) EFM response on a scan line over a PS particle of permittivity *ε*_p_ = 2.6 covered by an alumina shell of permittivity *ɛ*_i_ = 9.8. The signals are characterized by a single maximum. This confirms that, as predicted during the simulations presented in the previous sections, although the tip encounters different capacitive regions, EFM detects the particle and interphase assembly as one apparent particle with the same dimensions of the assembly while having a unique global effective permittivity. At the same lift height, the change in the interphase or alumina shell in this case, also changes the EFM signal amplitude related to a change of the equivalent capacitance. The signal in the center of particles with and without the interphase model was indeed sufficient to prove its existence.

#### 4.3.2. Interphase Detection without Matrix

*(i) Determination of the Actual Tip-Sample Distance*. The first step towards the quantification of the electrostatic response is determining the actual tip-sample distance during measurements. When the amplitude during the second scan is relatively low, the tip-sample distance is equal to *z* ≈ *z*_0_ + lift. *z*_0_ is the initial distance of the tip during the first scan, that is approximately equal to the amplitude of vibration during the topography scan since we use tapping mode. It is obtained by performing an approach curve of the amplitude of vibration over a stiff sample [18]. The lift height corresponds to the retracted height from topography during the second scan. Typical *z*_0_ working distances are 16 nm for PS samples and 13.5 nm for alumina films samples.

*(ii) Determination of the Actual Tip Radius*. Besides tip-sample distance determination, a precise calibration of the tip geometry (and size) is crucial for the quantification of electrostatic force gradients. Manufacturer only claims that *R*_0_ is less than 25 nm.

Hence, an *α*(*z*) curve has been performed on a bare metallic substrate for different tips of the same series. We fitted experimental results with simulations [15, 18]. In this case, knowing *z*, and since no dielectric film is present, the only fitting parameter becomes *R*_0_. The tip cone half-angle *Ø* has been fixed to 15° and the height *h* to 10 *μ*m.

**Figure 14:**
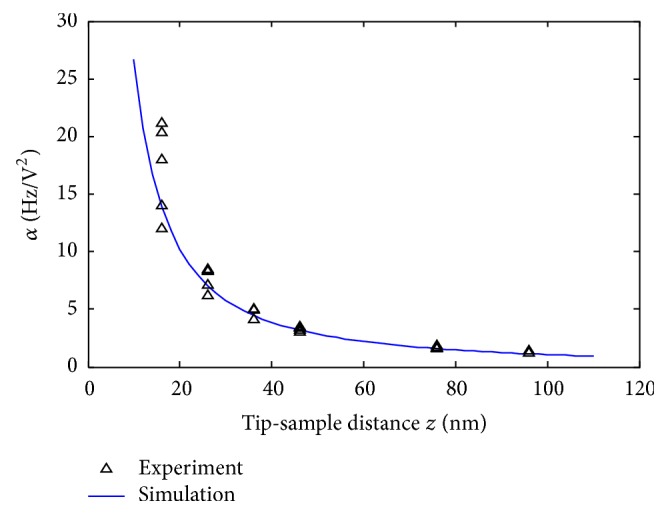
Best *R*_0_ fit curve between experimental results and simulation, obtained for a *R*_0_ = 13 nm with *Ø* = 15° and *h* = 10 *μ*m.

As shown in Figure 14, a tip radius of *R*_0_ = 13 nm fitted quite well most experimental curves. In the following, we will describe how we measured *α*(*z*) with simulations.

*(iii) Alpha Coefficient versus Alumina Thickness*. Comsol software only calculates the interaction force. In order to obtain the force gradient, we calculate the force *F* at different tip-sample distances *z*. Then, we calculate the first derivative of the resulting equation of *F*(*z*) to get the gradient *G*(*z*).

**Figure 15:**
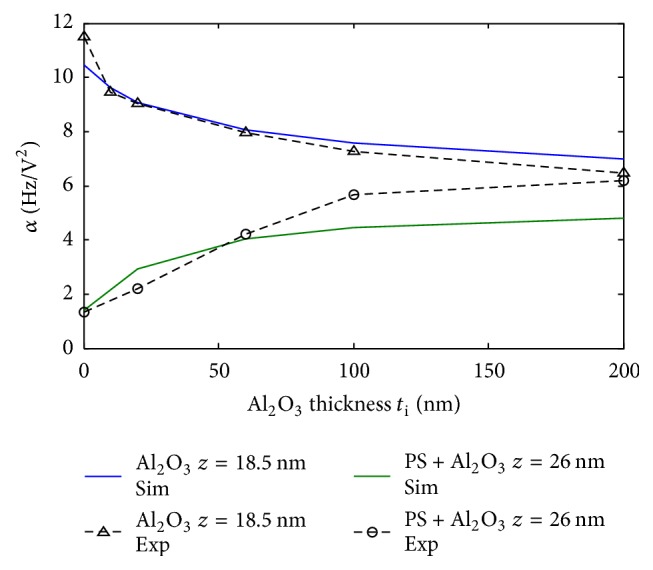
Experimental versus simulated *α* values for PS + Al_2_O_3_ (*z* = 26 nm) and Al_2_O_3_ alone (*z* = 18.5 nm) for different alumina thicknesses.

Our material geometry and permittivities have been modeled as presented in Figure 2. We fixed all parameters and changed the thickness of alumina shell *t*_i_. We present in Figure 15 EFM experimental and simulation results for PS + Al_2_O_3_ shells at *z* = 26 nm and for Al_2_O_3_ films alone at *z* = 18.5 nm for different alumina thicknesses *t*_i_. We can notice the same trend between simulations and experiments and a good agreement of *α* values between EFM measurements and our model. However, the fitting is more robust for Al_2_O_3_ films alone. Over PS + Al_2_O_3_ assembly, the fitting is less accurate especially at high shell thicknesses. We explain these differences by the fact that in the case of PS particles, of 250 nm approximate diameter, the interelectrode distance is already equal to 250 nm, and the addition of 100 or 200 nm alumina shell increases even further this distance. At these distances, the micrometric parts of the cantilever can start to have a nonnegligible effect on the interaction between the probe and the sample [36]. In this work, the precise geometry of the probe has not been considered, which is not within the scope of accuracy of this study. We mainly focused on the nanometric part of the probe (apex part + extremity of the cone) which possesses an angle *Ø* around 15°. This can explain the underestimation of the calculated force gradient.

## 5. Conclusions

We have performed a theoretical study concerning the interaction between an EFM probe and a nanodielectric with finite-element numerical simulations. The nanodielectric was specified by the geometry, dimensions, and the dielectric permittivity of its three components (particle, interphase, and matrix). A unique contrast was obtained on a scan line over the inclusion indicating that the probe detects the particle and interphase assembly in the matrix as one apparent particle having a global effective permittivity. We discussed next the detectability limits of the interphase. Critical interphase permittivities and thicknesses for interphase detection with EFM were calculated. Then, we prepared samples with known and controlled properties to model a nanodielectric and verified experimentally EFM capability to study the interphase. The atomic layer deposition method has been used to deposit alumina shells having controlled thickness over polystyrene (PS) nanoparticles of 230 nm approximate diameter placed on metallic substrates. We studied the sensitivity of EFM to the alumina layer of thicknesses going from 20 to 200 nm. We demonstrated the presence of alumina and we proved its intrinsic dielectric contribution to the EFM signal with both DC and AC-force gradient detection methods. The sensitivity to alumina films has been proven with the easy access DC-force gradient detection in addition to the 2*ω* component measurement. DC-force gradients have been appropriately treated in order to isolate the unique contribution of the capacitance on the signal. Furthermore, since alumina films thickness is not known, a simple but special signal analysis has been used in order to counteract topographic changes effects on the signal. Finally, simulations attested good agreement with experimental results showing the validity of our model to quantitatively interpret EFM signals and to improve our understanding of nanodielectrics and interphases investigation with EFM.

After having shown the sensitivity to different interphase shell thicknesses without matrix, future works will be mainly oriented towards solving the complete problem using EFM measurements and simulations, that is, the detection and quantification (thickness and permittivity) of alumina shells, when the sample is covered by a matrix.
